# The contemporary role of blood products and components used in trauma resuscitation

**DOI:** 10.1186/1757-7241-18-63

**Published:** 2010-11-24

**Authors:** David J Dries

**Affiliations:** 1 Regions Hospital, 640 Jackson Street, St. Paul, MN 55101 University of Minnesota, 420 Delaware Street SE, Minneapolis, MN 55455, USA

## Abstract

**Introduction:**

There is renewed interest in blood product use for resuscitation stimulated by recent military experience and growing recognition of the limitations of large-volume crystalloid resuscitation.

**Methods:**

An editorial review of recent reports published by investigators from the United States and Europe is presented. There is little prospective data in this area.

**Results:**

Despite increasing sophistication of trauma care systems, hemorrhage remains the major cause of early death after injury. In patients receiving massive transfusion, defined as 10 or more units of packed red blood cells in the first 24 hours after injury, administration of plasma and platelets in a ratio equivalent to packed red blood cells is becoming more common. There is a clear possibility of time dependent enrollment bias. The early use of multiple types of blood products is stimulated by the recognition of coagulopathy after reinjury which may occur as many as 25% of patients. These patients typically have large-volume tissue injury and are acidotic. Despite early enthusiasm, the value of administration of recombinant factor VIIa is now in question. Another dilemma is monitoring of appropriate component administration to control coagulopathy.

**Conclusion:**

In patients requiring large volumes of blood products or displaying coagulopathy after injury, it appears that early and aggressive administration of blood component therapy may actually reduce the aggregate amount of blood required. If recombinant factor VIIa is given, it should be utilized in the fully resuscitated patient. Thrombelastography is seeing increased application for real-time assessment of coagulation changes after injury and directed replacement of components of the clotting mechanism.

## Pathogenesis of Acute Coagulopathy After Trauma

### Historical Perspective

Hemorrhagic shock accounts for a significant number of deaths in patients arriving at hospital with acute injury[1,2]. Patients with uncontrolled hemorrhage continue to succumb despite adoption of damage control techniques and improved transport and emergency care. Coagulopathy, occurring even before resuscitation, contributes significantly to the morbidity associated with bleeding[3,4]. Recognition of the morbidity associated with bleeding and coagulation abnormality goes back to the work of Simmons and coworkers during the Vietnam conflict[5]. Even at that time, standard tests including prothrombin time (PT) and partial thromboplastin time (PTT) correlated poorly with acute resuscitation efforts. Similar work in the late 1970s was performed in civilian patients receiving massive transfusion. Again, PT, PTT and bleeding time were only helpful if markedly prolonged[6].

Lucas and Ledgerwood performed a variety of studies in large animals and patients to determine changes in the coagulation profile with hemorrhagic shock[7]. In patient studies, platelet count fell until 48 hours after injury and increased dramatically during convalescence. Bleeding times and platelet aggregation studies mirrored platelet levels. Reductions in fibrinogen, Factor V and Factor VIII were noted with hemorrhagic shock which normalized by day one after bleeding. By day four after bleeding, fibrinogen increased to supranormal levels. Clotting times mirrored fibrinogen, Factor V and Factor VIII levels. These investigators then studied the role of Fresh Frozen Plasma (FFP) supplementation in hemorrhagic shock with two studies. In animal studies, subjects received shed blood and crystalloid with some animals receiving Fresh Frozen Plasma. In this animal work, Fresh Frozen Plasma did not improve coagulation factors, fibrinogen and Factors II, V, VII and VIII. In a second controlled study, fresh frozen plasma was given not only during blood volume restoration but also for an additional hour during ongoing controlled hemorrhage without shock. Fresh Frozen Plasma prevented reduction in coagulation factors compared to animals not receiving fresh frozen plasma. Clotting times paralleled coagulation factor levels. From this work, Lucas and Ledgerwood ultimately concluded that hemorrhagic shock resuscitation requires restoration of blood loss with packed cells and crystalloid while FFP is appropriately added due to losses of coagulation proteins[7].

Studies in the 1970s and 1980s provided additional detail regarding the limitation of simple laboratory parameters and factor levels in evaluation of patient response to massive transfusion[6,8]. In a study of 27 patients requiring massive transfusion, platelet counts fell in proportion to the size of transfusion while Factors V and VIII correlated poorly with the volume of blood transfused. Where coagulopathy appeared, the majority of patients responded to platelet administration. In this early work, the most useful laboratory test for predicting abnormal bleeding was the platelet count. A falling fibrinogen level was felt to be indicative of DIC. The bleeding time, prothrombin time and partial thromboplastin time were not helpful in assessing the cause of bleeding unless they were greater than 1.5 times the control value[6]. In a subsequent series of studies from the same investigative group, 36 massively transfused patients were followed for microvascular bleeding. Moderate deficiencies in the clotting factors evaluated were common but they were not associated with microvascular bleeding. Microvascular bleeding was associated with severe coagulation abnormalities such as clotting factor levels less than 20% of control. In statistical analysis, clotting factor activities less than 20% of control were reliably reflected by significant prolongation of PT and PTT. These investigators also suggested that empiric blood replacement formulas available at the time were not likely to prevent microvascular bleeding because consumption of platelets or clotting factors did not consistently appear and simple dilution frequently did not correspond to microvascular bleeding[8].

The attention of the American trauma community was drawn to coagulopathy after trauma with description of the *"bloody vicious cycle" *by the Denver Health team over 20 years ago[3]. These investigators noted the contribution of hypothermia, acidosis and hemodilution associated with inadequate resuscitation and excessive use of crystalloid. Subsequent work extended these observations describing early coagulopathy which could be independent of clotting factor deficiency (consistent with scattered earlier observations)[9]. Moore and others, in a recent multicenter trial of hemoglobin oxygen carriers, observed early coagulopathy in the setting of severe injury, which was present in the field, prior to Emergency Department arrival and initiation of resuscitation. Coagulopathic patients were at increased risk for organ failure and mortality. One concern in the presentation of these patients was inconsistency in available laboratory data which identified patients at risk[10].

Dating to development of Advanced Trauma Life Support, trauma teams have used fixed guidelines for plasma and platelet replacement during massive transfusion to prevent and correct dilutional coagulopathy. Empiric plasma and platelet replacement was based on washout physiology, a mathematical model of exchange transfusion. The model assumes stable blood volume and calculates exponential decay of each blood component with bleeding. In severe injury, however, these assumptions may not apply: blood volume fluctuates widely and bleeding rates vary with blood pressure and replacement frequently lags behind blood loss. Replacement guidelines based on simple washout physiology may be inadequate[11-14].

In one of the first papers to question historical transfusion practice in the setting of massive trauma, Hirshberg, Mattox and coworkers, utilizing clinical data, developed a computer model designed to capture interactions between bleeding, hemodynamics, hemodilution and blood component replacement during severe hemorrhage. Replacement options were offered in the model and their effectiveness evaluated[11].

In the computer model, an intravascular compartment was created accepting crystalloid infusion and calculating the exchange of free water between intravascular and interstitial spaces. The basic compartment model was a *"leaky bucket" *where inflow is determined by a clinical scenario and outflow (bleeding rate) is proportional to systolic blood pressure. The effectiveness of crystalloid resuscitation decreases during massive hemorrhage in proportion to the volume of blood lost. In this computer simulation, an exponential model of effectiveness for crystalloid resuscitation is employed. Hemostasis was modeled by a relationship sensitive to blood pressure with 90 mmHg associated with ongoing bleeding and 50 mmHg associated with minimal blood loss. The impact of dilution on prothrombin time, fibrinogen and platelets were based on data obtained from dilution curves in the hospital coagulation laboratory from patients with significant hemorrhage. Standard product replacement quantities were assumed[11,15,16].

**Figure 1:**
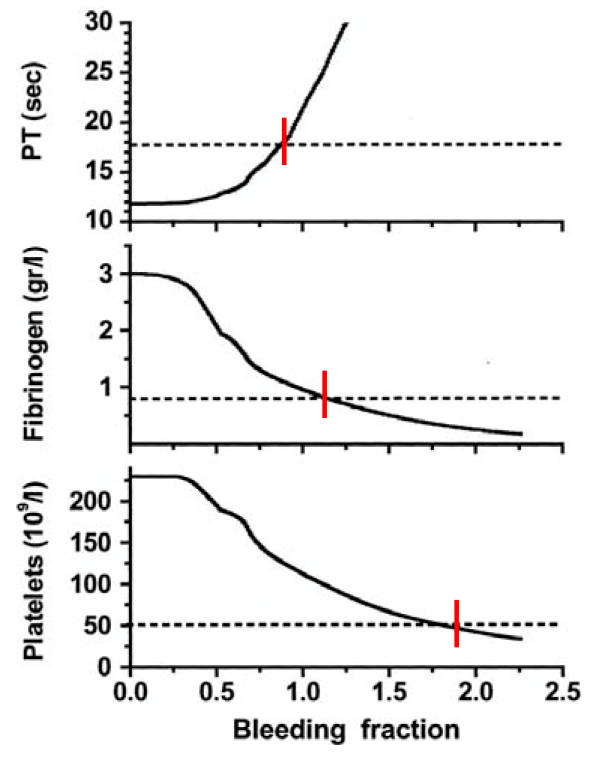
**Behavior of the computer model for massive bleeding without replacement of clotting factors or platelets**. Bleeding fraction is the volume of blood lost divided by the estimated blood volume (4,900 mL). Early loss of clotting factors is seen. (Dotted line is threshold for critical component deficit.)

After setting thresholds for acceptable loss of clotting factors, platelets and fibrinogen, the authors modeled behavior of coagulation during rapid exsanguination without clotting factor or platelet replacement. The prothrombin time reached a critical level first followed by fibrinogen and platelets. If patients were resuscitated with smaller amounts of crystalloid, leaving overall blood volume reduced, the effective life of components of the coagulation cascade was increased. More aggressive Fresh Frozen Plasma (FFP) replacement was indicated by this model. The optimal ratio for administration of FFP to packed red blood cells (PRBCs) in this analysis was 2:3. Delayed administration of FFP led to critical clotting factor deficiency regardless of subsequent administration of FFP. Fibrinogen depletion was easier to correct. Even after administration of 5 units of PRBCs, the hemostatic threshold for fibrinogen was not exceeded if a FFP to PRBC ratio of 4:5 was employed. Analysis of platelet dilution show that even if platelet replacement was delayed until 10 units of PRBCs were infused, critical platelet dilution was prevented with a subsequent platelet to PRBC ratio of 8:10[11] (Figure 1).

The essential message of this work is that massive transfusion protocols in existence when this study was performed provide inadequate clotting factor replacement during exsanguinating hemorrhage and neither prevent or correct dilutional coagulopathy.

### Acute Coagulopathy of Trauma

Brohi and coworkers from the United Kingdom helped to reinvigorate discussion of scattered seminal observations regarding coagulopathy after injury by adding new coagulation laboratory techniques to earlier clinical observations[17]. Reviewing over 1,000 cases, patients with acute coagulopathy had higher mortality throughout the spectrum of Injury Severity Scores (ISS). Contrary to historic teaching that coagulopathy was a function of hemodilution with massive crystalloid resuscitation, these authors noted that the incidence of coagulopathy increased with severity of injury but not necessarily in relationship to the volume of intravenous fluid administered to patients. Brohi and others helped to reemphasize the observation that acute coagulopathy could occur before significant fluid administration which was attributable to the injury itself and proportional to the volume of injured tissue. Development of coagulopathy was an independent predictor of poor outcome. Mediators associated with tissue trauma including humoral and cellular immune system activation with coagulation, fibrinolysis, complement and kallikrein cascades have since been associated with changes in hemostatic mechanisms in the body similar to those identified in the setting of sepsis[17-19,1].

MacLeod, in a recent commentary, discussed factors contributing to coagulopathy in the setting of trauma[20]. That hypothermia relates to development of coagulopathy has been demonstrated in vitro and in clinical studies. Temperature reduction impairs platelet aggregation and decreases function of coagulation factors in non-diluted blood. Patients with temperature reduction below 34°C had elevated prothrombin and partial thromboplastin times. Coagulation, like most biological enzyme systems, works best at normal temperature. Similarly, acidosis occurring in the setting of trauma as a result of bleeding and hypotension also contributes to clotting failure. Animal work shows that a pH <7.20 is associated with hemostatic impairment. Platelet dysfunction and coagulation enzyme system changes are noted when blood from healthy volunteers is subjected to an acidic environment[21,22].

We are now noting that with or without hypothermia and acidosis post-traumatic coagulopathy may develop in a significant number of patients. Possible explanations for this phenomenon include factor dilution, clotting system depletion and disseminated intravascular coagulation. Interplay of these and other factors in the face of ongoing blood loss is still not understood. Crystalloids and colloids can dilute available clotting factors. Increasing microvascular tissue injury may deplete the coagulation system due to demands of hemorrhage control at multiple sites. Third, and most interesting, loss of clotting factors associated with exaggerated inflammation is now being reported in association with injury. The presence of predictors of coagulopathy has been suggested by historical data from the United States and the European Union. While flaws exist in this preliminary epidemiologic data, it is now clear that coagulation changes after injury reflect more than the amount of crystalloid given[21-24].

Hess and coworkers as part of an international medical collaboration (The Educational Initiative on Critical Bleeding in Trauma) developed a literature review to increase awareness of coagulopathy independent of crystalloid administration following trauma[19]. The key initiating factor is tissue injury. This is borne out by original work demonstrating the close association between tissue injury and the degree of coagulopathy. Patients with severe tissue injury but no physiologic derangement, however, rarely present with coagulopathy and have a lower mortality rate[25,26]. Tissue damage initiates coagulation as endothelial injury at the site of trauma leads to exposure of subendothelial collagen and Tissue Factor which bind von Willebrand factor, platelets and activated Factor VII (FVII). Tissue Factor or FVII activate plasma coagulation and thrombin and fibrin are formed. A subsequent amplification process mediated by factor IX may take place on the surface of activated platelets[27].

Hyperfibrinolysis is seen as a direct consequence of the combination of tissue injury and shock. Endothelial injury accelerates fibrinolysis because of direct release of Tissue Plasminogen Activator[19,28]. Tissue Plasminogen Activator expression by endothelium is increased in the presence of thrombin. Fibrinolysis is accelerated because of the combined affects of endothelial Tissue Plasminogen Activator release due to ischemia and inhibition of Plasminogen Activator Inhibitor in shock. While hyperfibrinolysis may focus clot propagation on the sites of actual vascular injury, with widespread insults, this localization may be lost. Specific organ injuries have been associated with coagulopathy. Traumatic brain injury has been noted with increased bleeding thought due to release of brain-specific thromboplastins with subsequent inappropriate clotting factor consumption. Hyperfibrinolysis has also been seen in more recent studies of head-injured patients. Long bone fractures along with brain and massive soft tissue injury also may prime the patient for coagulopathy[29,30]. These contributing factors, however, are inadequate to lead to catastrophic coagulopathy if present in isolation.

A number of important cofactors must be present to stimulate coagulopathy in the setting of trauma[19]. Shock is a dose-dependent cause of tissue hypoperfusion. Elevated base deficit has been associated with coagulopathy in as many as 25% of patients in one large study. Progression of shock appears to result in hyperfibrinolysis. The exact processes involved are unclear. One mediator implicated in coagulopathy after injury is Activated Protein C. Immediate post-injury coagulopathy is likely a combination of effects caused by large volume tissue trauma and hypoperfusion.

**Figure 2:**
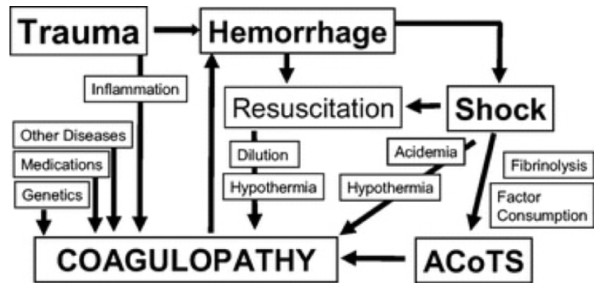
**Diagram showing some of mechanisms leading to coagulopathy in the injured**. ACoTS = Acute Coagulopathy of Trauma-Shock.

Several other historic factors are acknowledged for their contribution to coagulopathy after trauma. Hess and others continue to acknowledge the impact of dilution of coagulation factors with crystalloid resuscitation after injury[19]. While acknowledging inadequate clinical data at present, equivalent ratios of FFP, PRBCs and platelets must be considered for management of coagulopathy after injury. Hypothermia and acidemia are controlled to reduce their impact on enzyme systems[31]. Inflammation is receiving greater attention as a consequence of severe injury. Recent data suggests earlier activation of the immune system after injury than previously proposed. Similar to sepsis, cross-talk has been noted between coagulation and inflammation systems. Activation of coagulation proteases may induce inappropriate inflammatory response through cell surface receptors and activation of cascades such as Complement and platelet degranulation[32-34]. Trauma patients are initially coagulopathic with increased bleeding but may progress to a hypercoagulable state putting them at increased risk for thrombotic events. This late thrombotic state bears similarities with coagulopathy of severe sepsis and depletion of Protein C. Injured and septic patients share a propensity toward multiple organ failure and prothrombotic states. A diagram displaying the interrelated mechanisms contributing to coagulopathy after trauma is presented (Figure 2).

## Blood Component Therapy and the "Ratio"

Despite work from multiple groups suggesting that simple replacement of packed red blood cells was not a sufficient answer to the most severely injured patient, particularly in the setting of coagulopathy, the concept of combination blood component replacement remained outside the mainstream of trauma care for over 20 years[7,8,3]. In part, this may reflect the difficulty in characterizing coagulopathy after injury due to limitations of static testing as described above. It took additional conflicts in the Middle East and experience in a multinational group of trauma centers to bring awareness of the need for multiple blood component therapy in massive bleeding to the level of general trauma practice.

The 1970s and 1980s saw several groups propose resuscitation of significant hemorrhage with combinations of blood components. Kashuk and Moore proposed multicomponent blood therapy in patients with significant vascular injury[3]. In a study of patients with major abdominal vascular injury, Kashuk and coworkers noted frequent deviation from a standard ratio of 4:1 or 5:1 for units of packed red blood cells to units of Fresh Frozen Plasma. The ratio was 8:1 in nonsurvivors and 9:1 where overt coagulopathy was noted. Fifty-one percent of patients in this series were coagulopathic after vascular control was obtained. Using multivariate analysis, Ciavarella and coworkers from the Puget Sound Blood Center and Harborview Medical Center proposed aggressive supplementation of platelets in the setting of massive transfusion. These investigators noted that platelet counts below 50 × 10^9 ^per liter correlated highly with microvascular bleeding in trauma and surgery patients. Fibrinogen repletion was also emphasized. Other guides to resuscitation included fibrinogen level, prothrombin time and partial thromboplastin time. Supplemental Fresh Frozen Plasma or cryoprecipitate was recommended for low fibrinogen levels[8]. Lucas and Ledgerwood, summarizing extensive preclinical and clinical studies, suggested administration of Fresh Frozen Plasma after 6 units of packed red blood cells had been infused. Additional Fresh Frozen Plasma was recommended for every five additional packed red blood cell transfusions. Monitoring included platelet count, PT and PTT after each 5 units of packed red blood cells are administered. Platelet transfusion is generally unnecessary unless the platelet count falls below 50,000[7].

Despite this early work, blood loss continues to be the major cause of early death after injury accounting for 50% of deaths occurring during the initial 48 hours after hospitalization. Bleeding remains a common cause of preventable deaths after injury[35-37]. Many centers are beginning to establish protocols for massive transfusion practice but criteria and compliance continues to vary. Trauma centers are examining approaches to comprehensive hemostatic resuscitation as a replacement strategy for earlier approaches based on rapid, early infusion of crystalloids and PRBCs alone[17-20].

**Figure 3:**
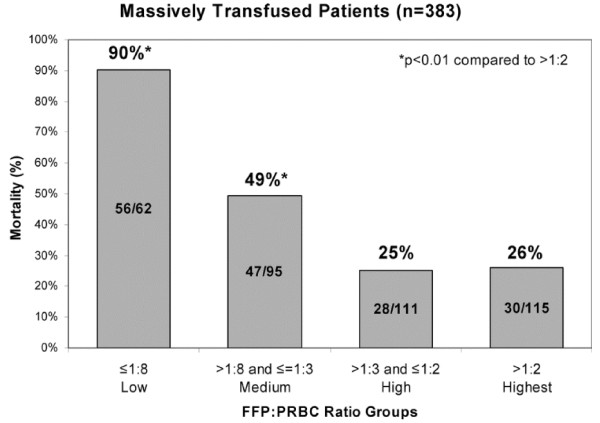
**Mortality Decrease with Higher FFP:PRBC Ratios**.

Rhee and coworkers, using the massive database of the Los Angeles County Level I Trauma Center, examined transfusion practices in 25,000 patients[38]. Approximately 16% of these patients received a blood transfusion. Massive transfusion (≥10 units of PRBCs per day) occurred in 11.4% of transfused patients. After excluding head-injured patients, these authors studied approximately 400 individuals. A trend toward increasing FFP use was noted during the six years of data which was reviewed (January 2000 to December 2005). Logistic regression identified the ratio of FFP to PRBC use as an independent predictor of survival. With a higher the ratio of FFP:PRBC, a greater probability of survival was noted. The optimal ratio in this analysis was an FFP:PRBC ratio of 1:3 or less. Rhee and coworkers provide a large retrospective dataset demonstrating that earlier more aggressive plasma replacement can be associated with improved outcomes after bleeding requiring massive transfusion. Ratios derived in this massive retrospective data review support the observations of Hirshberg, Mattox and coworkers[11]. Like the data presented by Kashuk and coworkers in another widely cited report, this retrospective dataset suggests improved clinical outcome with increased administration of FFP[39] (Figure 3).

Another view of damage control hematology comes from Vanderbilt University Medical Center in Nashville, Tennessee. This group implemented a Trauma Exsanguination Protocol involving acute administration of 10 units PRBC with 4 units FFP and 2 units platelets. In an 18 month period, 90 patients received this resuscitation and were compared to a historic set of controls. The group of patients receiving the Trauma Exsanguination Protocol as described by these investigators had lower mortality, much higher blood product use in initial operative procedures and higher use of products in the initial 24 hours though overall blood product consumption during hospitalization was decreased[40].

**Figure 4:**
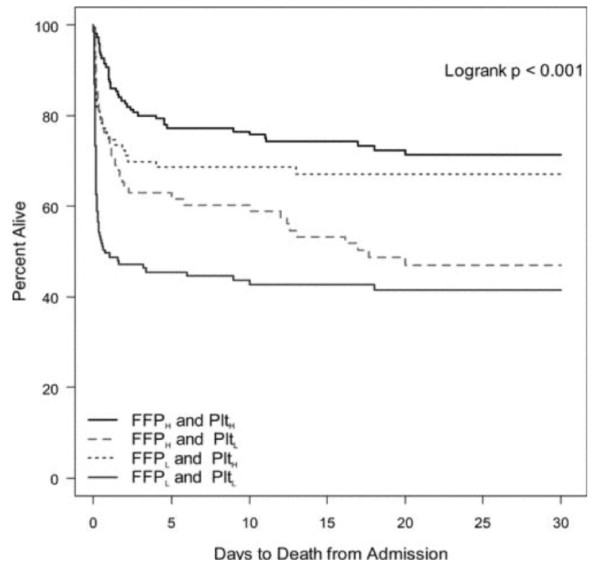
**30-day survival using Kaplan-Meier curves comparing patients receiving high ratios of fresh frozen plasma (FFP) and platelets to PRBCs versus patients receiving low ratios of either FFP or platelets**. Patients with best outcomes had high ratios of both FFP and platelets to PRBCs while worst outcomes came with low ratios of both FFP and platelets to PRBCs. Where one component, either FFP or platelets was low, intermediate outcomes were obtained.

The strongest multicenter civilian data examining the impact of plasma and platelet administration along with red blood cells on outcome in massive transfusion comes from Holcomb and coworkers[41]. These investigators report over 450 patients obtained from 16 adult and pediatric centers. Overall survival in this group is 59%. Patients were gravely ill as reflected by an admission base deficit of -11.7, pH 7.2, Glasgow Coma Score of 9 and a mean Injury Severity Score of 32. Examination of multicenter data reflects an improvement in outcome as the ratio of Fresh Frozen Plasma to packed red blood cells administered approaches 1. Fresh Frozen Plasma, however, is not the sole solution to improved coagulation response in acute injury. These workers also examined the relationship of aggressive plasma and platelet administration in these patients. Optimal outcome in this massive transfusion group was obtained with aggressive platelet as well as plasma administration. Worst outcomes were seen when aggressive administration of plasma and platelets did not take place. Where either FFP or platelets were given in higher proportion in relationship to packed red cells intermediate results were obtained. Not surprisingly, the cause of death which was favorably affected was truncal hemorrhage. Examination of the Kaplan-Meier curves provided by these workers demonstrates that the impact of early blood product administration on mortality is seen in improved outcomes immediately after injury (Figure 4).

A summary statement comes from Holcomb and a combination of military and civilian investigators[18,19]. These workers identify a patient group at high risk for coagulopathy and resuscitation failure due to hypothermia, acidosis, hypoperfusion, inflammation and volume of tissue injury. In the paradigm proposed by these writers, resuscitation begins with prehospital limitation of blood pressure at approximately 90 mmHg preventing renewed bleeding from recently clotted vessels. Intravascular volume resuscitation is accomplished using thawed plasma in a 1:1 or 1:2 ratio with PRBCs. Acidosis is managed by use of THAM and volume loading with blood components as hemostasis is obtained. These workers utilize rFVIIa *"occasionally" *along with early units of red cells. A massive transfusion protocol for these investigators included delivery of packs of 6 units of plasma, 6 units of PRBC, 6 units of platelets and 10 units of cryoprecipitate in stored individual coolers. These coolers are continued until notification comes from the trauma team. Even in causalities requiring resuscitation with 10-40 units of blood products, Holcomb and coworkers found that as little as 5-8 liters of crystalloid are utilized during the first 24 hours representing a decrease of at least 50% compared to standard practice. The lack of intraoperative coagulopathic bleeding allows surgeons to focus on surgical hemorrhage. The goal is arrival of the patient in ICU in a warm, euvolemic and nonacidotic state. INR approaches normal and edema is minimized. Subjectively, patients treated in this way are more easily ventilated and easier to extubate than patients with a similar blood loss treated with standard crystalloid resuscitation and smaller amounts of blood products. Clearly, these clinical observations warrant development of hypothesis-driven research. Holcomb and others suggest that massive transfusion will be required in 6-7% of military practice and 1-2% of civilian trauma patients.

An intriguing evaluation of the relationship of blood product administration to mortality comes from the Alabama School of Medicine in Birmingham[42]. Again, patients requiring massive transfusion defined as >10 units PRBCs within 24 hours were studied. One hundred thirty-four individuals met this definition between 2005 and 2007. This study, however, defined FFP:PRBC ratios in two ways; first, as a fixed value at 24 hours and then as a time varying covariate. High ratio was defined as >1:2 with low ratio as <1:2 units of FFP:PRBCs. Using 24 hour mortality comparison, patients with a high ratio of FFP:PRBCs administered had a significant improvement in outcome. As is the case in other studies of massive transfusion, mortality occurred early in hospital course.

In a telling second analysis, the Alabama investigators examined temporal mortality among low and high ratio patient groups[42]. During early time intervals, most deaths occurred in the group receiving a low ratio for that interval while during the later time intervals more deaths occurred in the group receiving a high FFP:PRBC ratio. The pattern of mortality in this data includes the potential for survival bias as the majority of deaths occurred when most patients resided in the low ratio group, before the accumulation of patients in the high ratio group. These investigators then performed Cox regression modeling with FFP:PRBC ratio as a time dependent coordinate. In this assessment, the survival advantage associated with the high ratio group as demonstrated previously disappeared. Adjustment for platelet, cryoprecipitate and rFVIIa administration did not change this result. Because many deaths, those associated with hemorrhage, occurred early in the hospital course, many patients in these time intervals were in the low ratio group (low FFP use) rather than the high ratio group. Survival bias was introduced as patients in the low ratio group died early which fixed them at a low FFP:PRBC ratio and prevented them from transitioning to the high ratio group. These observations are also reflected in a paper from the Stanford group by Riskin and coworkers. Riskin and others identified improved outcomes with ***rapid ***administration of blood products to appropriate patients even if equivalent amounts of FFP and PRBCs were employed[43]. This important analysis of retrospective data reinforces the need for carefully orchestrated prospective studies.

### Complications of Massive Transfusion

There are many clinical issues beyond component "ratios" for the injured patient.

#### TRALI

While summary data suggests that increased use of plasma and platelets may improve outcome in the setting of massive transfusion, use of these additional components should be done thoughtfully[44-47]. A growing body of work describing Transfusion-Related Acute Lung Injury (TRALI) identifies early and late respiratory failure secondary to this problem as the major complication of transfusion. The likelihood of TRALI increases with plasma-based products; thus, Fresh Frozen Plasma and platelets may place patients at increased risk. At present, we can only provide supportive care for the patient with TRALI, though use of fresh products may reduce the risk of late TRALI which appears to be a storage lesion. We must also be aware that giving packed red cells, platelets and plasma in a 1:1:1 ratio does not replace fresh whole blood which may be the optimal blood product for resuscitation. In a recent review, Sihler and Napolitano point out that administration of stored components in a 1:1:1 ratio provides reduced amounts of red cells, clotting factors and platelets relative to fresh whole blood. FFP, however, may provide secondary benefit as a fibrinogen source[45,47,48].

#### Transfusion Risks May Be Increased With "Old" Blood

Modern blood banking is based on component therapy. Blood components undergo changes during storage which may affect the recipient including release of bioactive agents with immune consequences. Generation of inflammatory mediators is related to duration of unit storage. Small datasets note an increased risk of multiple organ failure where the age of units of transfused blood is increased. Thus, fresh blood may be the most appropriate initial resuscitation product for trauma patients requiring transfusion[49-52].

Other age-related changes of stored blood have been identified. For example, red cell deformability is reduced not only after injury but in stored blood as the duration of storage increases. Supernatants from stored red blood cells have been documented to prime inflammatory cells in vitro and induce expression of adhesion molecules in neutrophils and proinflammatory cytokines. Among proinflammatory cytokines identified are IL-6, IL-8 and TNF-α. Finally, with increased length of red blood cell storage, free hemoglobin concentrations in red cell products are increased. Free hemoglobin in units of stored red blood cells can bind nitric oxide and cause vasoconstriction. Local vascular effects related to the vasoconstrictive properties of stored red blood cells may limit off-loading of oxygen to tissues, the principle rationale for transfusion[49,50].

#### What is the Effect of Giving Uncross-matched Blood?

Many centers initiate blood product resuscitation with uncross-matched blood. Lynn and coworkers have examined their clinical experience with administration of uncross-matched type-O red blood cells[53]. This product is given at the discretion of attending physicians to patients with active hemorrhagic shock and need for immediate transfusion before the availability of cross-matched blood. Frequently, the decision for giving uncross-matched type-O PRBCs is a subjective assessment based on vital signs, physical examination and experience. In a review of over 800 patients from a five year period, approximately 3,000 units of uncross-matched type-O blood were given. The mean Injury Severity Score in the patients receiving this blood was 32. The univariate analysis based on amount of uncross-matched type-O blood demonstrated a linear correlation between the number of units given and the probability of death. Obviously, quantity of uncross-matched type-O blood given is also a surrogate for depth of shock, rate of hemorrhage and is a marker for mortality due to injury. These observations were confirmed by Inaba and coworkers who examined use of over 5,000 uncross-matched units over six years. Administration of uncross-matched blood was indicative of the need for massive transfusion and higher mortality[54].

### When Should We Employ a Massive Transfusion Protocol?

Little is written about the criteria for activation of a massive transfusion protocol. In our trauma center, we use the classification of shock, secondary to hemorrhage, promoted by the *American College of Surgeons *and the *Advanced Trauma Life Support *(ATLS) program[55]. Patients presenting with persistent hypotension in conjunction with other signs of Class III shock are candidates for administration of our massive transfusion protocol. Repeated determination of vital signs and the appropriate clinical setting is necessary to trigger the massive transfusion protocol. Despite using this time-honored set of criteria, many patients who do not require massive transfusion may be started on this protocol. We clearly need better criteria to determine initiation of a massive transfusion protocol. As noted above, historical data and recent reports from the military, suggest that in the military setting, 6-7%% of patients will require massive transfusion, and in the civilian setting, only 1-2% of patients will require massive transfusion[18].

A recent analysis from the German Trauma Registry examined parameters available within the first 10 minutes after hospital admission as predictors of the need for massive transfusion[56]. Massive transfusion was defined in this analysis as administration of at least 10 units of PRBCs during the initial phase of therapy. The result was a simple scoring system called TASH (Trauma-Associated Severe Hemorrhage) using hemoglobin (2-8 points), base excess (1-4 points), systolic blood pressure (1-4 points), heart rate (2 points), free fluid on abdominal ultrasound (3 points), open and/or dislocated fractures of extremities (3 points), pelvic fracture with blood loss (6 points) and male gender (1 point). A score of 15 points in the TASH Scale predicts a 50% risk of massive transfusion. Lynn suggests that similar indicators emerged in a review of the Miami Trauma Registry[53].

**Figure 5:**
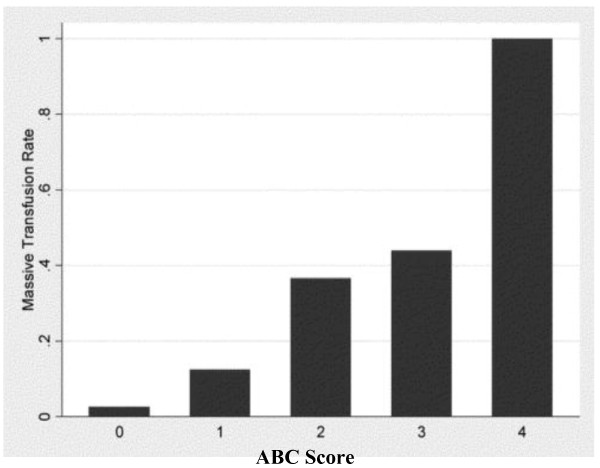
**Rate of Massive Transfusion by ABC Score**.

**Figure 6:**
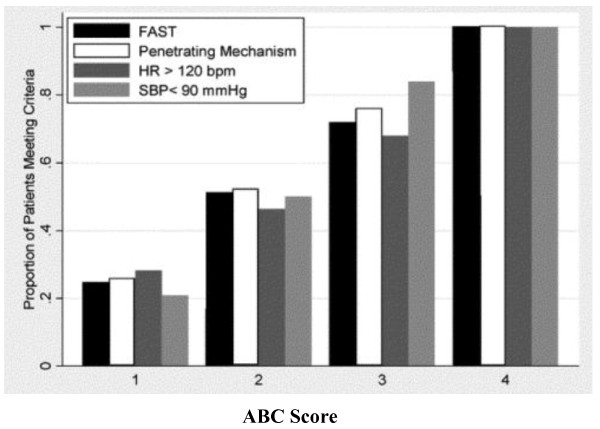
**Individual contribution of each component of ABC Score to the likelihood of massive transfusion**.

Cotton and the group at Vanderbilt in the United States propose a similar predictive score reflecting the need for massive transfusion in trauma[57]. These authors identify four dichotomous components available at the bedside of injured patients early in evaluation. The presence of any one component contributes one point to the total score for a possible range of scores from 0 to 4. Parameters include penetrating mechanism (0 = no, 1 = yes); Emergency Department systolic blood pressure of 90 mmHg or less (0 = no, 1 = yes); Emergency Department heart rate of 120 beats/min or greater (0 = no, 1 = yes); and positive abdominal sonogram (0 = no, 1 = yes). When all of these factors are present, the Nashville group suggests that the likelihood of massive transfusion is very high (Figure 5). Examination of contribution from individual components to the ABC (Assessment of Blood Consumption) Score of these investigators reveals that each contributes in roughly equal proportion (Figure 6). In a second multicenter study, Cotton and coworkers validated the ABC Score with data obtained from Parkland Hospital in Dallas, the Johns Hopkins Institutions in Baltimore and a dataset for Vanderbilt University. The predictive value of the ABC Score was consistent across the three trauma centers examined. In fact, the negative predictive value was 97% across this trial. From this data, the authors argue that less than 5% of patients who will require massive transfusion will be missed using the ABC Score[58].

In another recent study, Cotton and coworkers evaluated the ability of uncross-matched blood transfusion in the Emergency Department to predict early (<6 hours) massive transfusion of red blood cells and blood components. Massive transfusion was defined as the need for 10 units or more of packed red blood cells in the first six hours. Early massive transfusion of plasma was defined as six units or more of plasma in the first six hours. Early massive transfusion of platelets was defined as two or more apheresis platelet transfusions in the first six hours. These authors studied 485 patients who received Emergency Department transfusions and 956 patients who did not receive Emergency Department transfusions after trauma. Patients receiving uncross-matched red blood cells in the Emergency Department were more than three times more likely to receive early massive transfusion of red blood cells. These authors recommend considering Emergency Department transfusion of uncross-matched red blood cells as a trigger for activation of an institution's massive transfusion protocol[59].

#### What is a Massive Transfusion Protocol?

Massive transfusion is most commonly defined as administration of ten units of packed red blood cells in the first 24 hours after admission to hospital. Generally, this does not include emergency department uncross-matched products. Cotton, Holcomb and coworkers define massive transfusion of plasma as the administration of six units or more in the first 24 hours after admission. Massive transfusion of platelets is defined as the transfusion of two or more apheresis units in the first 24 hours after admission. These workers distinguish between "early" massive transfusion and massive transfusion in recent writings. Early massive transfusion of red blood cells is defined as transfusion of ten units or more of packed red blood cells in the first six hours after admission. Early massive transfusion of plasma is defined as administration of six units of plasma or more in the first six hours after admission. Early massive transfusion of platelets is defined as transfusion of two or more apheresis units in the first six hours after admission. In defining massive transfusion and early massive transfusion in this way, the authors address the time bias which may be associated with the pattern of blood product administration and attempt to distinguish between the patient requiring therapy for early emergent bleeding as opposed as to the patient requiring ongoing stabilization with blood product administration[59].

### Role of Recombinant Factor VIIa

Recombinant FVIIa (rFVIIa) was introduced in the 1980s as a hemostatic agent[60]. Recombinant FVIIa is thought to act locally at the site of tissue injury and vascular wall disruption by injury with presentation of Tissue Factor and production of Thrombin sufficient to activate platelets. The activated platelet surface can then form a template on which rFVIIa can directly or indirectly mediate further coagulation resulting in additional thrombin generation and ultimately fibrinogen conversion to fibrin. Clot formation is stabilized by inhibition of fibrinolysis due to rFVIIa-mediated activation of Thrombin Activatable Fibrinolysis Inhibitor. Initially, rFVIIa was used in patients with congenital or acquired hemophilia and inhibiting antibodies toward factor VIII or IX and it has been licensed in the United States and other parts of the world for this purpose. There is significant off-label use of rFVIIa in surgical applications including uncontrolled bleeding in the operating room or following injury.

Other recent investigations suggest that rFVIIa acts by binding activated platelets and activating Factor Xa on platelet surface independent of its usual co-factor, Tissue Factor. The activation of Factor X (FX) on the platelet surface would normally be via the FIXa-FVIIIa complex which is deficient in hemophilia. Factor Xa produces a *"burst" *of thrombin generation required for effective clot formation. At high doses, rFVIIa can partially restore platelet surface FX activation and thrombin generation[61,62].

Until recently, much of the literature associated with rFVIIa comes from case reports or uncontrolled series. In fact, a literature review published in 2005 by Levi and coworkers identified publications with rFVIIa noted until July, 2004. The majority of publications were case reports or case series. Twenty-eight clinical trials represented 6% of publications. Eleven of the clinical trials addressed the needs of hemophiliacs, three trials reflected patients with other coagulation defects while seven trials were devoted to patients with liver disease. Only one study at the time of this review was conducted in surgical patients. Thus, much of the work of the trauma community with rFVIIa is recent and the number of studies is small[63,64].

Physiologic limits for the use of rFVIIa in the setting of injury are being identified[65]. Meng and coworkers examined the effectiveness of high dose rFVIIa in hypothermic and acidotic patients. This group studied blood collected from healthy, consenting adult volunteers. For temperature studies, blood reactions with rFVIIa were kept at 24°C, 33°C and 37°C. For pH studies, the pH of the reaction was adjusted by solutions of saline buffered to obtain the desired pH. In temperatures studies, rFVIIa activity on phospholipids and platelets was not reduced significantly at the 33°C compared to 37°C. In all, the activity of rFVIIa and Tissue Factor was reduced by approximately 20% at 33°C in comparison to 37°C. However, a physiologic pH decrease from 7.4 to 7.0 reduced the activity of rFVIIa with Tissue Factor by over 60%. These observations are consistent with clinical data, reviewed below, suggesting reduced efficacy of rFVIIa in the setting of acidosis.

The largest clinical data set with regard to management of trauma comes from Boffard and the NovoSeven Trauma Study Group[66,67]. These investigators, in a prospective, randomized trial, enrolled 301 patients of whom 143 patients with blunt trauma and 134 patients with penetrating trauma were eligible for analysis. Examination of the primary endpoint, red blood cell transfusion requirements during the initial 48 hour observation period after the initial dose of study drug, reveals that administration of rFVIIa in the setting of blunt trauma significantly reduced 48 hour red blood cell requirements by approximately 2.6 units. The need for massive transfusion was reduced from 20 of 61 patients in the placebo group to 8 of 56 patients in the group receiving rFVIIa. In patients with penetrating trauma, no significant effect of rFVIIa was observed with respect to 48 hour red blood cell transfusion requirements with an aggregate red blood cell reduction of approximately one unit over the study course. The need for massive transfusion in penetrating trauma was reduced from 10 of 54 patients in the placebo group to 4 of 58 patients with rFVIIa. No difference between treatment groups was observed in either blunt or penetrating trauma patient populations with respect to administration of FFP, platelets or cryoprecipitate. Despite the reduced need for massive transfusion, there was no difference in mortality in either the blunt or penetrating trauma groups.

There are three additional multicenter trials reporting use of rFVIIa in injured patients[68-70]. Raobaikady and others examined blood product use in 48 patients treated for pelvic fractures. The rFVIIa dose employed was 90 μg/kg and the primary outcome examined was perioperative blood loss during reconstruction. No difference was noted in comparison to patients receiving placebo. In the recently reported CONTROL Trial, Hauser and coworkers, in a randomized prospective format, studied 573 patients[69]. The majority of these individuals sustained blunt trauma. Protocol administration for factor VII and initial trauma care were carefully employed. In patients with both penetrating and blunt trauma, rFVIIa reduced blood product use but did not affect mortality compared with placebo. Thrombotic events were similar across study groups. This trial was stopped early because of lack of efficacy for rFVIIa demonstrated on interim statistical analysis. The largest clinical experience with rFVIIa comes from the United States military[70]. Wade and others recently reviewed experience with over 2,000 soldiers. A subset of this group, 271 patients, was matched by epidemiologic criteria to injured soldiers who did not receive rFVIIa. Fifty-one percent of patients in each group received massive transfusion. There was no difference in complications or mortality with administration of rFVIIa (Table 1).

**Table 1:** Summary of Important Trials Published*

Author and Year	Patient Group	rFVIIa Dosing	Primary Endpoint	Outcomes
Boffard; [67] J Trauma 2005; 59:8-18	Penetrating and blunt trauma (301)	200+100+100 μg/kg	RBC units first 24 hours	Reduction in RBCs (blunt)

Raobaikady; [68] Br J Anaesth 2005; 94:586-591	Pelvic fractures (48)	90 μg/kg	Perioperative blood loss	No difference

Hauser; [69] J Trauma 2010; 69:489-500	Blunt and penetrating trauma (573)	200+100+100 μg/kg	Mortality, blood product use	No mortality difference, Less product use

Wade; [70] J Trauma 2010; 69:353-359**	Military trauma (2,050)	Varied	Complications, mortality	No difference

*Modified from Ann Emerg Med 2009; 54:737-744.

**Large retrospective case control analysis.

The largest reported single center North American experience with rFVIIa comes from the Shock Trauma Institute at the University of Maryland[71]. In this retrospective study, experience with 81 coagulopathic trauma patients treated with rFVIIa during the years 2001 to 2003 is compared with controls matched from the Trauma Registry during a comparable period. A number of causes for coagulopathy were noted. The largest group of patients (46 patients), suffered acute traumatic hemorrhage. Traumatic brain injury (20 patients), warfarin use (9 patients) and 6 patients with various hematologic defects including 2 individuals with FVII deficiency were included in this review. Coagulopathy was reversed, based on clinical response in 61 of 81 cases. Significant reduction in prothrombin time was seen in patients receiving rFVIIa. Overall mortality in the patients receiving rFVIIa was 42% versus 43% in a group of patients identified as coagulopathic with comparable injuries and lactate levels identified from the Trauma Registry. In comparing patients who appeared to be responders to non-responders to rFVIIa, the Maryland group noted poorer outcomes in acidotic patients consistent with previous preclinical work. These authors did note a small number of severely acidotic patients who did survive with administration of rFVIIa. Thus, simple acidosis may warrant reconsideration if use of rFVIIa is otherwise appropriate. The only thrombotic complications observed in this series, segmental bowel necrosis in 3 patients with mesenteric injury after rFVIIa therapy, was also seen in 2 individuals who did not receive rFVIIa.

One additional recent trial in hemorrhagic stroke is worthy of comment. Eight hundred and forty-one patients with intracerebral hemorrhage were randomized to placebo, low dose or high dose rFVIIa within 4 hours of onset of stroke. Endpoints studied were important; disability and death. Low dose rFVIIa was 20 μg/kg body weight and high dose rFVIIa was 80 μg/kg body weight. While scheduled follow-up CT scans demonstrated reduced volume of hemorrhage in patients receiving rFVIIa, no difference in functional outcome or mortality was identified. Serious thromboembolic events were similar in all three groups. Arterial adverse events were more frequent in the high dose rFVIIa group than in placebo (9% versus 4%, *p *= 0.04). Adverse events were closely followed. The frequency of elevated troponin I values was 15%, 13% and 22% and the frequency of ST elevation myocardial infarction was 1.5%, 0.4% and 2.0% in the placebo group and the groups receiving 20 μg and 80 μg of rFVIIa per kilogram respectively. CT evidence of acute cerebral infarction was identified in 2.2%, 3.3% and 4.7% of patients in the placebo group and the groups receiving 20 μg and 80 μg of rFVIIa per kilogram respectively. Age was identified as a risk factor for thromboembolic events in a post hoc analysis. rFVIIa is cost effective but has not changed outcomes in traumatic brain injury in a more recent trial[72].

Most concerning in recent discussion regarding the use of rFVIIa in the setting of injury is a potential role for this material in magnifying early traumatic coagulopathy. Administration of rFVIIa in supraphysiologic doses may increase combined activity of Thrombin and Thrombomodulin. Within the coagulation cascade, Thrombomodulin from endothelium complexes with Thrombin in association with activation of Protein C and its cofactor Protein S. Through consumption of Plasminogen Activator Inhibitor I, fibrinolysis is increased and Tissue Plasminogen Activator is also released by endothelium in shock states contributing to fibrinolysis (discussed above). In addition to effects just listed, increased binding of Thrombin to Thrombomodulin reduces conversion of Fibrinogen to Fibrin and platelet activation. If, therefore, in the setting of hypoperfusion, administration of rFVIIa increases Thrombin production, additional activation of Protein C (with coagulopathy) may occur rather than generation of Fibrin. Administration of rFVIIa in the setting of hypoperfusion may contribute to rather than control coagulopathy[1,60].

Two recent metaanalyses also suggest a cautious approach[73,74]. Hsia and others conclude that the use of rFVIIa may reduce the need for blood transfusion and possibly reduce mortality[73]. The dose of rFVIIa should be limited to 90 μg/kg and an increased risk of arterial thrombosis may exist. A more pessimistic view comes from Hardy and two coauthors in a recent review from the *Annals of Thoracic Surgery*[74]. These workers conclude that generalized use of rFVIIa to prevent or control bleeding in nonhemophiliac patients cannot be recommended[66,74].

### Newer Products

#### Prothrombin Complex Concentrate

Currently, Fresh Frozen Plasma (FFP) is the standard choice to correct coagulopathy after major injury. Drawbacks associated with FFP such as the need for thawing and the requirement for ABO compatibility may be limited by holding thawed plasma or administering Type AB or Type A plasma in emergencies. These resources may only be available in major trauma centers. A more readily available and concentrated coagulation factor replacement such as Prothrombin Complex Concentrate (PCC) could provide advantages in emergent situations[75-77]. In addition to factor VII, PCC contains coagulation factors II, IX and X and the anticoagulation proteins C and S. PCC in combination with fibrinogen has been shown to enhance coagulation and final clot strength in a porcine model of dilutional coagulopathy[78]. More recent work using controlled splenic injury and hemodilution demonstrates more rapid hemostasis and augmented thrombin generation in comparison to rFVIIa. Notably, time to splenic hemostasis was not significantly reduced by rFVIIa in comparison to placebo[79].

#### Tranexamic Acid

Part of the response to surgery and trauma is clot breakdown (fibrinolysis), which may become pathological in the setting of injury. Antifibrinolytic agents reduce blood loss in patients with both normal and exaggerated fibrinolytic response to surgery and do so without apparent increase in postoperative complications[80-82].

Tranexamic acid is a synthetic derivative of the aminoacid lysine which inhibits fibrinolysis by blocking the lysine binding sites on plasminogen. Fifty-three studies including 3,836 participants have involved tranexamic acid in patients undergoing elective surgery. Tranexamic acid reduced the need for blood transfusion by a third in these patients with no significant reduction in mortality. Tranexamic acid was recently investigated as a means to reduce blood product utilization and mortality in trauma patients[83,29,36,84].

In a massive randomized, control trial spanning 40 countries, over 20,000 adult trauma patients with or risk of significant bleeding were randomly assigned within eight hours of injury to either tranexamic acid (loading dose 1 gram over 10 minutes and then infusion of 1 gram over 8 hours) or matching placebo[84]. Randomization was balanced by center and participants and study staff were blinded to treatment allocation. The primary outcome was death in hospital within four weeks of injury described with complications including bleeding, vascular occlusion, multiorgan failure, traumatic brain injury and others. All cause mortality was significantly reduced with tranexamic acid (14.5%) in comparison to placebo (16.0%). The risk of death specific to bleeding was also significantly reduced (4.9% with tranexamic acid vs 5.7% with placebo). This is by far the largest outcome study related to bleeding in the setting of injury. The use of tranexamic acid is supported by this large dataset. Remarkably, there were no adverse events regarded as serious, unexpected, or suspected to be related to the study treatment. Even more important, the results of this trial were not dependent on the results of laboratory tests. Study admission was based on clinical criteria. One can speculate that administration of this material guided by appropriate laboratory testing might lead to even stronger support for its use.

The authors freely admit that this trial provides limited insight into the mechanism by which tranexamic acid reduces the risk of death in bleeding patients after injury. Previous workers have demonstrated, however, that hyperfibrinolysis is a frequent feature of coagulopathy after injury and raise the possibility that antifibrinolytic agents such as tranexamic acid might operate via this mechanism. Unfortunately, this trial did not measure fibrinolytic activity. Finally, the authors note that additional work is required to determine if tranexamic acid is beneficial in the setting of traumatic brain injury.

## Monitoring of Coagulopathy

Up to 25% of multiple trauma patients suffer from coagulopathy. Coagulopathy may be associated with hemodilution, transfusion of blood products, hypothermia, acidosis and shock. As Fresh Frozen Plasma, coagulation factors and other pharmacologic therapies are administered, it is of great value to monitor the effects of these interventions on coagulation. The current standard of care for coagulation assessment is a series of tests including prothrombin time expressed as international normalized ratio (INR), activated partial thromboplastin time (APTT), thrombin time (TT) and platelet counts. This monitoring is often flawed because of differences between laboratory conditions in the clinical environment together with significant intervals between drawing of blood and obtaining results which may render these tests useless[85,86].

One approach to this problem would be to improve point of care monitoring of coagulation using the technique of thrombelastography (TEG). TEG offers the advantage of providing a real-time graphic representation of clot formation and whole blood. Unlike standard laboratory tests, TEG offers analysis of the whole coagulation cascade permitting identification of depleted components and directed therapy to correct coagulopathy. The procedure involves placing a small volume of blood in an oscillating cup at 37°C or at patient temperature. As the blood in the cup clots, the motion of the cup as rotated is transmitted to a pin dipped in the blood. TEG has been used in preliminary studies to evaluate changes in coagulation in injured patients[85-88].

Carroll and coworkers evaluated a TEG system and platelet mapping, which can also be performed using a TEG technology, and correlated these values with transfusion and fatality in a series of trauma patients. Initial blood samples in this study were obtained at accident scenes and in the Emergency Department. Overall, little difference was seen in TEG parameters between the accident scene and Emergency Department. Standard TEG parameters and the platelet mapping assay employed did not correlate with the need for transfusion except in patients where poor platelet function was identified. However, abnormality in TEG parameters and platelet mapping studies were strongly correlated with mortality. In this respect, TEG and platelet mapping parameters were more sensitive than standard clotting tests such as PT, aPTT and platelet count[89].

Thrombelastography (TEG) may also facilitate detection of hypercoagulable states. In an ICU study of burned and traumatized patients, Park and coworkers found a significant number of non-bleeding injured patients developed a hypercoagulable state within the initial days after injury. In comparison of TEG to PT and aPTT, TEG demonstrated increased coagulation while PT and aPTT did not. Despite aggressive thromboprophylaxis in patients followed during this study, 3 of 58 patients suffered pulmonary emboli[90].

As discussed in an excellent review by Kashuk, Moore and others, TEG was first described in 1948[1,91]. It assesses clot strength from the time of initial fibrin formation to clot retraction ending in fibrinolysis. TEG is the only single test providing information on the balance between the opposing components of coagulation, thrombosis and lysis while the battery of traditional coagulation tests, which include bleeding time, PT, aPTT, thrombin time, fibrinogen levels, factor assays, platelet counts and functional assays are based on isolated, static data points[92,93]. TEG examines interaction of the entire clotting cascade and platelet function in whole blood. PT measures only the extrinsic clotting system while aPTT examines an enzymatic reaction in the intrinsic clotting cascade. Hypothermia, a common complication of injury also affects the coagulation process and leads to functional abnormalities. Platelet dysfunction is influenced by thrombin and fibrinogen concentrations and can be affected by hypothermia, acidosis and hypocalcemia[1]. Much of the recent experience with TEG comes from Europe. Some European centers use the ROTEM device which differs from classic TEG in that the blood specimen is stationary while the pin is rotated instead of the cup. Like TEG, ROTEM has been useful in providing global evaluation of the coagulation process including fibrinolysis[94].

A variant of TEG reported by the Denver group is rapid Thrombelastography (rTEG). rTEG differs from conventional TEG in that Tissue Factor is added to the whole blood specimen allowing a more rapid coagulation reaction and subsequent evaluation. A recent European report also suggests that rTEG is useful in evaluation of patients after injury[1,88,23].

**Figure 7:**
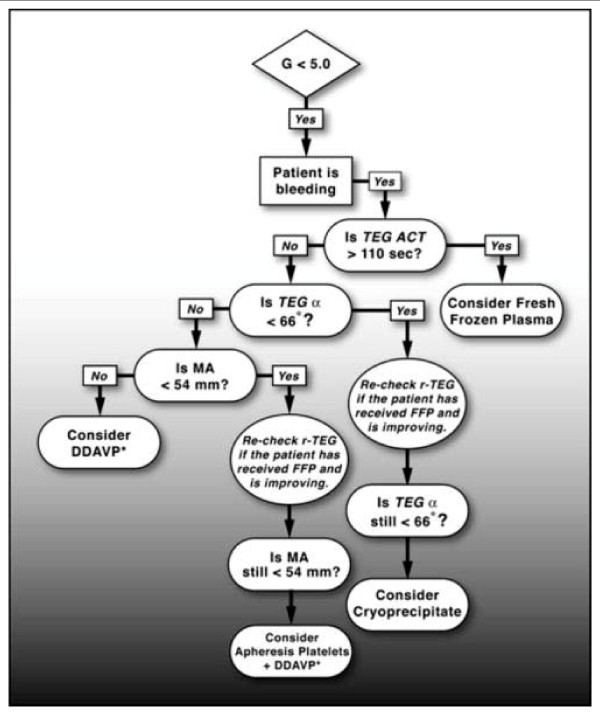
**Denver rTEG Protocol - G is a computer-generated value reflecting the complete strength of the clot from initial fibrin burst through fibrinolysis and is calculated from amplitude which begins at the bifurcation of the tracing**. This is based on a curvilinear relationship: G = (5,000 × amplitude)/(100 minus amplitude). Conceptually, G is the best measure of clot strength as it reflects the contributions of the enzymatic and platelet components of hemostasis. Normal coagulation is defined as G between 5.3 and 12.4 dynes/cm^2^.

The most sophisticated North American program of blood component resuscitation guided by rTEG has been developed by investigators in Denver[1]. The Denver group uses component infusion therapy based on rTEG findings. They anticipate use of FFP to provide a final ratio of 1:2 to 1:3 units of FFP to Packed Red Blood Cells and propose that goal-directed therapy using rTEG facilitates stepwise correction of coagulation abnormalities by comparative assessment of serial rTEG tracings (Figure 7). A particular benefit of this approach is identification of fibrinolysis which may be treated with epsilonaminocaproic acid. Hyperfibrinolysis may also be identified with ROTEM technology. The Denver protocol is depicted based on a series of rTEG measures[94,88,23].

Two recent European consensus statements reflect on the dilemma of monitoring blood component therapy in the setting of resuscitation. Gaarder and coworkers in the ***Scandinavian Guidelines - "The Massively Bleeding Patient" ***suggest a relationship between administration of FFP and red cell products given the dose adjustment by laboratory measurement of fibrinogen, coagulation parameters and by thrombelastography[95]. In the setting of uncontrolled bleeding, recommended administration of plasma is in a 1:1 ratio with red cell products with guidance by the parameters described above. These authors further acknowledge limitation of conventional coagulation assays to describe the dynamic bleeding condition of injured patients[96,97]. TEG is, therefore, recommended by the group as a whole blood analysis providing quantitative information regarding hemostasis and changes occurring in coagulation response during product infusion. These writers hold TEG superior with regarding to identification of clinically relevant coagulopathy and as a predictor of the need for product administration in trauma patients[87].

A more conservative stance is found in the recent European Guideline (***Management of Bleeding Following Major Trauma: An Updated European Guideline)***. Rossaint and the authors of this guideline recommend routine measure of INR, aPTT, fibrinogen levels and platelet counts. They also suggest that TEG be performed to assist in characterizing coagulopathy and in guiding hemostatic therapy[98].

The updated European guideline notes little evidence supporting optimal hemostatic monitoring tools in the setting of bleeding with trauma[98]. INR and aPTT monitor only the initiation of blood coagulation and represent a small fraction of thrombin production. Thus, conventional coagulation screens may be normal while overall blood coagulation is abnormal. Authors of the European consensus statement acknowledge TEG as a means to provide more complete monitoring of blood coagulation and fibrinolysis. Case series using TEG as reviewed by these authors have mixed results. Some authors utilize TEG to guide resuscitation with early platelet and Fresh Frozen Plasma administration and suggest improved outcomes. Other work demonstrates poor correlation between TEG and conventional coagulation parameters (however, this may be appropriate). Another possible approach is more frequent measurement of coagulation parameters with identification of trends which may predict coagulation outcomes after injury[99,85,100].

## Conclusion

Our understanding of the coagulopathy of trauma has changed significantly in recent years. In the setting of under perfusion and significant volume of tissue injury, coagulation abnormality may occur before fluid administration contrary to historical teaching which emphasizes hemodilution in the setting of massive crystalloid resuscitation. Development of early coagulopathy after trauma is an independent predictor of poor outcome. Growing recognition of early coagulopathy after injury has led to renewed emphasis on early blood product administration in the injured patient with bleeding[101,102].

While much important work has been done, we have more questions than answers in this area[103]. A number of simple observations can be made. Hemorrhage is still a common factor in the majority of patients sustaining early mortality after trauma[35]. Early use of blood products decreases the use of blood[47]. Criteria to identify patients appropriate for blood product administration are being developed[56,57]. The most promising of these criteria are the TASH Score from German investigators and the ABC Score from Cotton and coworkers. We continue to investigate the optimal combination of blood component therapy. In civilian practice, however, a ratio of packed red cells, Fresh Frozen Plasma and platelets of 1:1:1 is not equivalent to fresh whole blood, a clinical gold standard[44,47] (Table 2). Most investigators now agree that ratios of red blood cell units to plasma units should be no more than 2:1 to 3:1. Platelets must also be given but the dose varies with collection technique. An apheresis unit from one blood bank may be equivalent to several platelet "packs" from another source. Finally, rapid use of massive transfusion in appropriate patients is important.

**Table 2:** Whole Blood Composition Compared with Component Therapy

Whole Blood (500 mL)	Component Therapy (660 mL)
Hematocrit 38-50%	1 unit PRBC = 335 mL with hematocrit 55%

Platelets 150-400 K/μL	1 unit platelets = 50 mL with 5.5 × 10^10 ^platelets

Plasma Coagulation Factors = 100%	1 unit plasma = 275 mL with 80% of the coagulation activity compared with whole blood

1 unit PRBCs + 1 unit platelets + 1 unit FFP = 660 mL with hematocrit 29%, platelets 88 K/μL and coagulation activity 65% compared with whole blood. *(Component volumes reported are based on data provided from a North American reference.)*

Reproduced with permission from Chest 2010; 137:209-220.

The limitations of static clotting parameters and factor levels to characterize bleeding are now better recognized. TEG, ROTEM and rTEG offer real-time multifactorial evaluation of the clotting response to injury. Whether these new techniques also improve our ability to provide hemostatic resuscitation is unclear[102].

## Competing interests

The author declares that they have no competing interests.

## Author information

David J. Dries, MSE, MD, FACS, FCCM, FCCP is the Assistant Medical Director of Surgical Care for HealthPartners Medical Group and Division Head for Surgery at Regions Hospital, the Level I Trauma and Burn Center, in St. Paul, Minnesota, USA. He is also Professor of Surgery, Professor of Anesthesiology and Clinical Adjunct Professor of Emergency Medicine at the University of Minnesota. Dr. Dries also holds the *John F. Perry, Jr. Chair of Trauma Surgery *at the University of Minnesota.
